# Why Homeodynamics, Not Homeostasis?

**DOI:** 10.1100/tsw.2001.20

**Published:** 2001-04-04

**Authors:** David Lloyd, Miguel A. Aon, Sonia Cortassa

**Affiliations:** Microbiology (BIOSI), Cardiff University, P.O. Box 915, Cardiff, CF10 3TL, Wales, UK

**Keywords:** organised complexity, dynamic organisation, hemeodynamics, coherence, ultradian and circadian clocks, cytoskeleton, metabloic fluxes

## Abstract

Ideas of homeostasis derive from the concept of the organism as an open system. These ideas can be traced back to Heraclitus. Hopkins, Bernard, Hill, Cannon, Weiner and von Bertalanffy developed further the mechanistic basis of turnover of biological components, and Schoenheimer and Rittenberg were pioneers of experimental approaches to the problems of measuring pool sizes and dynamic fluxes. From the second half of the twentieth century, a biophysical theory mainly founded on self-organisation and Dynamic Systems Theory allowed us to approach the quantitative and qualitative analysis of the organised complexity that characterises living systems. This combination of theoretical framework and more refined experimental techniques revealed that feedback control of steady states is a mode of operation that, although providing stability, is only one of many modes and may be the exception rather than the rule. The concept of homeodynamics that we introduce here offers a radically new and all-embracing concept that departs from the classical homeostatic idea that emphasises the stability of the internal milieu toward perturbation. Indeed, biological systems are homeody- namic because of their ability to dynamically self-organise at bifurcation points of their behaviour where they lose stability. Consequently, they exhibit diverse behaviour; in addition to monotonic stationary states, living systems display complex behaviour with all its emergent characteristics, i.e., bistable switches, thresholds, waves, gradients, mutual entrainment, and periodic as well as chaotic behaviour, as evidenced in cellular phenomena such as dynamic (supra)molecular organisation and flux coordination. These processes may proceed on different spatial scales, as well as across time scales, from the very rapid processes within and between molecules in membranes to the slow time scales of evolutionary change. It is dynamic organisation under homeodynamic conditions that make possible the organised complexity of life.

## TABLE OF CONTENTS

**Introduction** ........ 134
**The Steady State** ........ 134
**The Oscillatory States in Biological Systems** ........ 135
**Dynamic Organisation under Homeodynamic Conditions** ........ 136
**A Geometric Interpretation of Homeodynamics** ........ 136
**Coherence** ........ 136
**Mechanisms of Coherence** ........ 138
Bottom-up Mechanisms ........ 138
Top-Bottom Mechanisms ........ 140
**Conclusions and Outlook** ........ 140
**References** ........ 143

## INTRODUCTION

In the second half of the last century, fundamental information about the spatio-temporal organisation in living systems became established. For the first time, we have at hand a biophysical theory to approach the quantitative and qualitative analyses of the organised complexity that characterise living systems.

Two main foundations of this biophysical theory are self-organisation[1,2], and Dynamic Systems Theory[3,4]. Self-organisation is deeply rooted in nonequilibrium thermodynamics[5] and the kinetics of nonlinear systems whereas Dynamic Systems Theory lies within the geometric theory of dynamical systems created by Poincaré[6,7].

By applying this biophysical theory of biological organisation to successively more complicated systems (i.e., artificial, artificial-biological-oriented, or biological),[8,9] it became clear that self-organisation is a fundamental and necessary property of living systems. Conditions under which self-organisation appears are[9]

- openness to fluxes of energy and matter;
- the operation of coupled processes through some common intermediate; and
- the occurrence of at least one process that exhibits a kinetic nonlinearity.

The mainstream of biological thought is only slowly recognising the dynamic nature of living systems. A main reason is that dynamics is a property of the whole system, integrated by manifold interactions in the form of parallel, sequential, and branched pathways of chemical reactions involving mass, energy, and information transfer. The type (i.e., negative or positive) and number of feedbacks nested in these mass-energy-information-carrying networks determine the dynamic behaviour of living systems by providing the necessary nonlinearities and coupling between processes. Emergent properties are accounted for in those interlinked circuits with autocatalytic potential[5,9,10,11]. These emergent properties are crucial in the transfer and processing of information in biochemical as well as cellular networks[12,13] that may be modulated either by genetic or epigenetic mechanisms.

## THE STEADY STATE

Hopkins'[14] description of life as the "expression of a dynamic equilibrium in a polyphasic system" provided several generations of biochemists with a model for thinking about mechanisms whereby stability can be achieved by balancing supply to demand. The "constancy of the internal environment" had already become an all-pervading concept in physiology ever since its introduction by Claude Bernard in 1865; this work has been translated[15]. Cannon[16] stressed that no organism can be considered in isolation from its environment and that because organisms exchange matter and energy with their surroundings, the process of homeostasis, whereby "steady states" are obstinately preserved and re-established, does not imply something set and immobile. The ideas of the organism as an open system were further developed into a "general systems theory" by von Bertalanffy from 1932[17] who built on the classical fascination of Heraclitus with the idea that life processes have features in common with a flowing stream, and of Roux, that a candle flame with its fluxes of matter and energy may also serve as an analogue. The thermodynamics of living organisms became of great interest in mainstream physiology in the 1930s and produced work of fundamental significance[18, 19].

Measurement of turnover rates of proteins had to await the discovery of deuterium and the heavy isotope of nitrogen. With these new metabolic labels, Schoenheimer[20] was able to show that the apparent stability of essential constituents belies an extremely dynamic reality. Post-war availability of radioisotopic carbon-containing compounds made these studies more easily feasible. Rittenberg[21] was able to show that the apparent overall absence of reactions in living cells simply indicates a balance that is subtly attained and maintained. It became clearly evident for the first time that "the approach to equilibrium is a sign of death."

The introduction of continuous culture techniques by Novic and Szilard[22] and by Monod[23] further entrenched these ways of thinking about the living state and led to the rather simplistic concept of "balanced growth."

Even in the late 1970s, there was an almost universal tendency among biochemists to overlook the growing evidence for the truly astonishing dynamic nature of life processes. For instance, at that time there was a great resistance to the idea that protein turnover is, for the most part, extremely rapid, and that even newly synthesised proteins are unstable and are, to a surprising degree, susceptible to degradative hydrolysis[24,25,26,27].

More recently, the essential roles of proteolytic systems in cellular economy[28] have become recognised to be so all-embracing that it has become evident that degradation is central, even to biological growth processes[26]. Turnover times *in vivo*, never easily measured, must be on a time scale of minutes and hours[29,30], rather than days, as formerly thought[31,32]. Even so, homeostasis is even now regarded as an universal principle; however, it becomes increasingly evident that maintaining the "status quo" cannot explain biological complexity and the long sequence of evolutionary changes that have led to its development.[10,11,33]

## THE OSCILLATORY STATES IN BIOLOGICAL SYSTEMS

In any system built without specially designed constraints, it is to be expected that oscillations will occur. This is true of mechanical, electrical, optical, and chemical systems. Thus it is so for the motions of boats and bridges, of electrons in circuits, of photons in lasers, and of molecules in test-tube mixtures. The more complex the system, the greater the numbers of degrees of freedom, and the greater the likelihood of outputs of varied frequencies and amplitudes. Processes that occur in nanoseconds are characteristic of the very small (e.g., vibrational events in molecules), whereas those that are slow, for instance, those that take billions of years (e.g., geological changes), are also on a giant scale. Time structure of living organisms also spans many scales (Fig. 1), from those occurring within single molecules and on membranes (measured in femtoseconds) to the slow evolutionary changes that require thousands of generations. Periodic processes can occur on all these time scales (Fig. 1).

The natural environment also has an obvious time structure that is generated by the geophysical cycles. Finely honed to optimise performance, organisms have evolved in periodic surroundings—the ebb and flow of the tides and the certainty that night will follow day, under the lunar cycle, throughout the seasons and the years. The matching of organism with environment has generated one of life's most fundamental characteristics: its rhythmic nature. The complex and varied time structure stems from this. Oscillations have been harnessed to provide rhythms, and where these rhythms become embedded so as to become heritable and to provide anticipatory advantages, they become biological clocks[34,35].

Although the existence of an endogenous clock has been recognised since de Mairan's observations in 1728 of the daily leaf movements in plants, the elements that comprise the time frame for living organisms have only slowly been discovered.

In biochemical systems, the oscillatory state was not observed until 1957, when Dysens and Amesz[36] showed os-

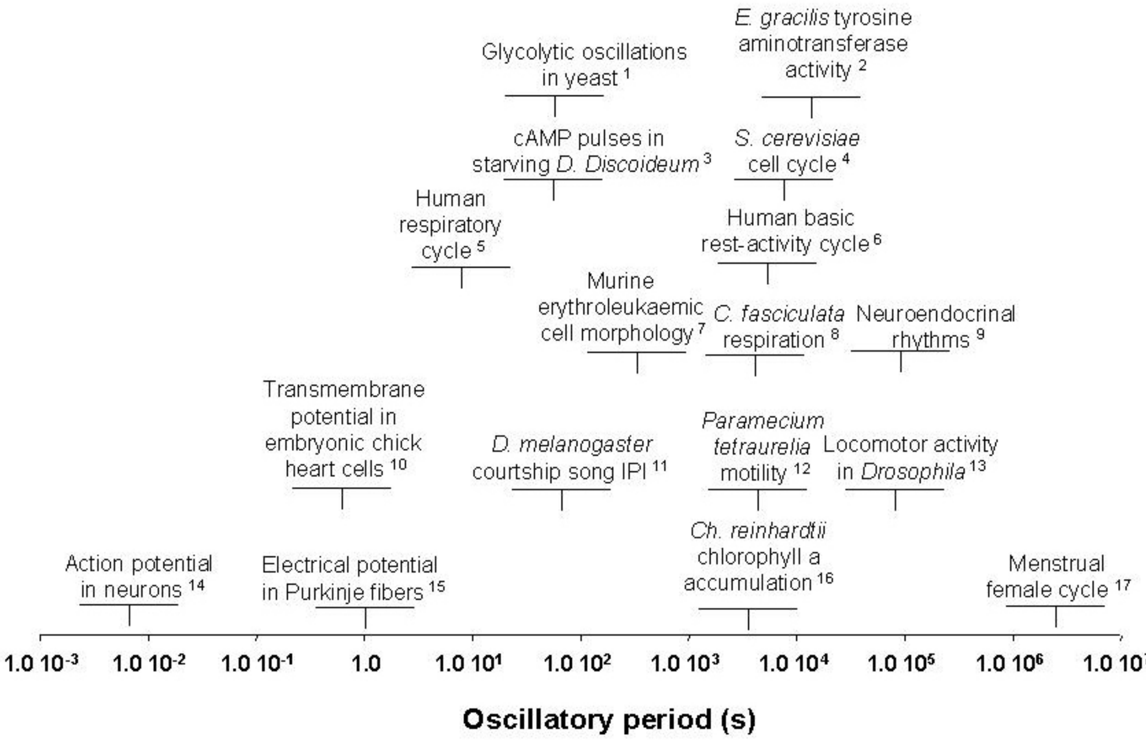

**FIGURE 1.** Periodic phenomena across several temporal scales. Ultradian clocks in the temporal domain between half to one hour with several intracellular outputs, coordinate (top-bottom) cell function and circadian, clock-type, behaviour. This coordination is an expression of *dynamic organisation under homeodynamic conditions*, according to the graphic visualisation depicted in Figures 2 and 3. The *x*-axis displays the period (in seconds) of the oscillatory phenomena, plotted in a logarithmic scale. The phenomena range from milliseconds (action potential in neurons) to a month (the female menstrual cycle). Numbers indicate references as follows: 1[44], 2[65], 3[66], 4[67], 5[68], 6[69], 7[70], 8[71], 9[72,] 10[73], 11[74], 12[75], 13[76], 14[77], 15[78], 16[79], and 17[8].

cillations in nicotinamide nucleotide levels in intact cells. The discovery of oscillations in glycolysis in yeast[37] led to an explosion of interest in the mechanism of control of glycolysis: This area has produced a wealth of data, even if the functions of this mode of metabolic operation are still not definitely understood. Fundamental theoretical treatments of observed kinetic data[38,39] established the importance of feedback inhibition and feedforward activation in the generation of oscillatory behaviour in the glycolytic pathway. The prediction of oscillatory dynamics in biosynthetic pathways[40] clearly demonstrated in synchronous cultures[29] was a further prescient step forward in the series of advances that concluded with a burgeoning recognition that complex dynamics is an integral property of life. Biophysical and biochemical oscillators are part of the spectrum of biological periodicities that even extend into geological time[8,9,41].

## DYNAMIC ORGANISATION UNDER HOMEODYNAMIC CONDITIONS

The concept of *dynamic organisation* has been introduced to describe function in cellular, or even supracellular, systems that arises as the spatio-temporal coherence of events resulting from the intrinsic, autonomous, dynamics of biological processes[9].The concept of *homeostasis* refers to the relative constancy, i.e., stable steady states, of the internal milieu's physiological status. The resistance to change by a homeostatic state is given by its stability to perturbations, i.e., given a stimulus and a transient response, it returns to its pre-stimulation state. In this sense, the concept of *homeodynamics* that we introduce here offers a radically new departing concept. Biological systems are homeodynamic because intracellular processes through their dynamic self-organisation may exhibit not only monotonic states (fixed points), but also a capacity for bistable switching threshold phenomena, waves, gradients, mutual entrainment, and periodic as well as chaotic behaviour. This complex behaviour is also evidenced in cellular phenomena such as dynamic (supra) molecular organisation and flux coordination (Fig. 2, 3).

At bifurcation points, a dynamic system loses stability and behavioural changes occur[1,42].These may be quantitative, qualitative, or both (Fig. 3). Quantitatively, it may happen that the system dynamics moves at limit points, to a different branch of steady-state behaviour (lower or higher), e.g., as in bistability. Under these conditions, the system does not change its qualitative behaviour, i.e., it continues to be at a point attractor, either a stable node or a focus. However, at some bifurcation points, drastic qualitative changes occur; the system evolves from a monotonic operation mode toward periodic (Hopf bifurcation) or chaotic motions (Fig. 3) [4,6,7,9,42,43,44,45].Thus, dynamically organised phenomena are *homeodynamic* (Fig. 2, 3), and visualised as demonstrating spatio-temporal coherence.

## A GEOMETRIC INTERPRETATION OF HOMEODYNAMICS

*Homeodynamics* refers to the continuous transformation of one dynamical system into another through instabilities at bifurcation points (Fig. 3). Generically, the state space is an n-dimensional space representing the set of all possible states of the system (n is the number of variables). The phase space is a two-dimensional state space which, filled with trajectories, constitutes the phase portrait of the system whose motion may be seen as a fluid flowing around itself (Fig. 3). When all the trajectories settle or approach a restricted region of the phase space, that region is called an attractor. An attractor has a basin to which a large number of trajectories tend over time (i.e., the dynamics tends to this attractor as time approaches infinity). Since various initial conditions are implicated, the set of points whose probability is given by its relative area or volume is called the basin of attraction (Fig. 3). A system has a set of basins of attraction and attractors that represents the integrated dynamic behaviour of the variables in the system as they mutually influence one another.

A dynamic system usually has basins with one attractor in each. Considering that the state space is decomposed into a set of basins, the system's motion may "flow" between attractors. The continuous motion of the system's dynamics and its potentiality for shifting between attractors at bifurcation points, based on its intrinsic dynamic properties, is what we call *homeodynamics* (Fig. 3). The latter is given by the general tendency of the system to self-organise and, in particular, because of nonlinear kinetic mechanisms as well as the dynamic coupling between processes (see above and Fig. 4). Thus, under homeodynamic conditions, a dynamic system may shift between attractors.

Dynamic systems that exhibit large attractors are more likely to behave homeodynamically than those with small attractors. In large attractors, "essential" variables are likely to be subjected to a large range of variation, rather than remaining clamped to small variations as in the case of small attractors (see below). Thus, we regard homeodynamics as a more general, all-embracing concept from which homeostasis becomes a special case.

## COHERENCE

Under *homeodynamic* conditions a system (e.g., network of reactions or cells) may exhibit emergent spatio-temporal coherence, i.e., dynamic organisation (Fig. 2, 4). Coherence may be understood as the synchronisation in space and time of molecules, or the architecture of supramolecular or supracellular structures, through self-organisation, in an apparent, "purposeful", functional way. Under coherent behavioural conditions, spatially distant (e.g., cytoplasmic)

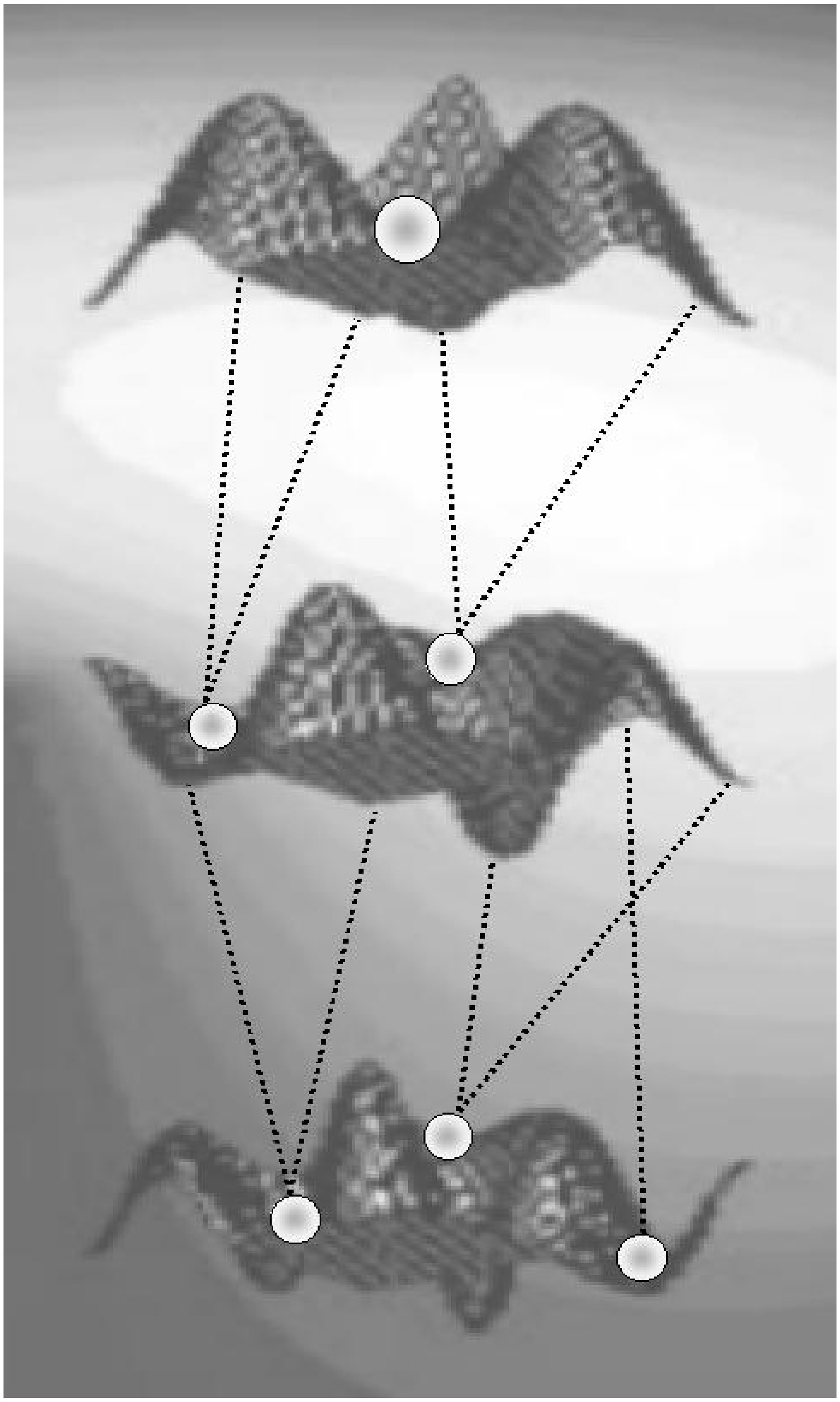

**FIGURE 2.** A graphic analogy of the concept of dynamic organisation under homeodynamic conditions. *Dynamic organisation* in, e.g., cells or tissues, is an emergent property arising from transitions between levels of organisation at bifurcation points in the dynamics of biological processes. The different landscapes represent the dynamic trajectories of (sub)cellular processes (e.g., enzyme activity, synthesis of macromolecules, cell division; indicated as spheres in the plot), resulting from the functioning of those processes at different spatio-temporal scales (levels of organisation). The dotted lines that link the spheres (different (sub)cellular processes) indicate the coupling between them. The coupling between processes that function simultaneously on different spatio-temporal scales *homeodynamically* modifies the system trajectories (the landscapes' shapes), as represented by the "motion" of the spheres through peaks, slopes, and valleys. The sphere on the landscape on top symbolises a process occurring at a higher level of organisation (higher spatial dimensions and lower relaxation times), i.e., a macroscopic one belonging to spatial structures (waves, macromolecular networks, subcellular organelles, etc.). Indeed, the functioning of the system is coordinated and coupling occurs top-bottom as well as bottom-up. The interdependent and coupled cross-talk between both flows of information trans-influences levels of organisation, i.e., beyond but through each level.

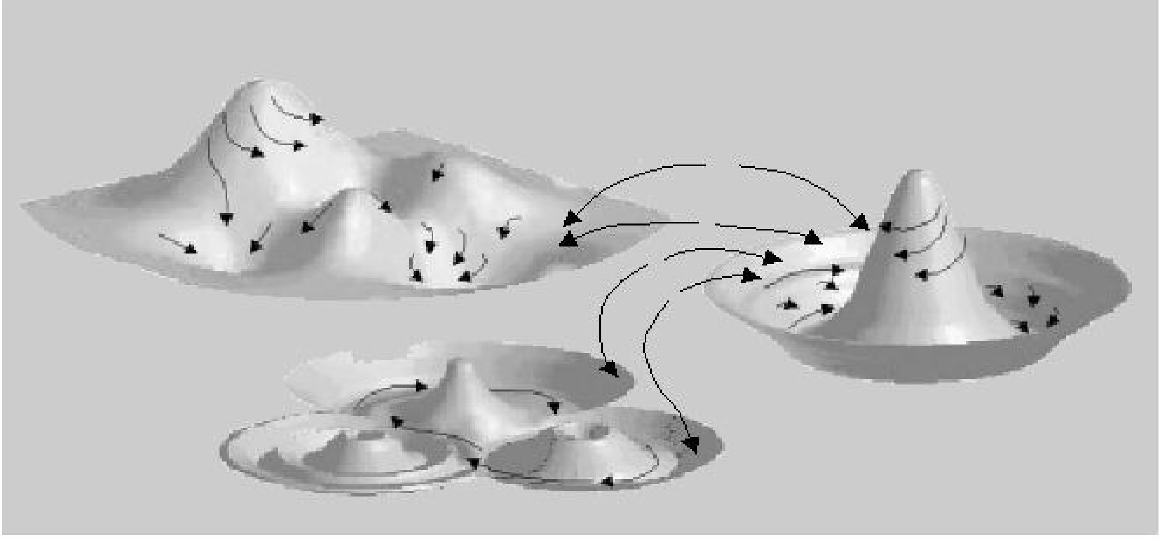

**FIGURE 3.** A geometric interpretation of *homeodynamics*. Several types of attractors with their corresponding basins of attraction are represented. The putative trajectories followed by system's dynamics, are emphasised by arrows as well as the separatrices between basins. The *homeodynamic condition* implies that the system's dynamics, visualised as a fluid flowing around itself, may shift between attractors at bifurcation points where stability is lost. Thereby, the system's dynamics, following a perturbation, flies away toward another attractor exhibiting either qualitative or quantitative changes in its behaviour. The upper left three-dimensional (3D) plot shows saddle and fixed points; the latter with different values, each one representing a different branch of steady states. Alternative occupancies of these states, following the change of a bifurcation parameter, give a bistable switch with memory-like features. Also stable and unstable foci are depicted in the upper left 3D plot. The lower right 3D plot, shows a limit cycle with its basin of attraction that may be attained through an unstable focus, characteristic of oscillatory behaviour (self-sustained or damped oscillations, respectively). The middle 3D plot depicts an attractor with three orbits embedded in it, with the potential for chaotic behaviour.

regions function simultaneously and coordinately, spanning spatial coordinates higher than the molecular or supramolecular realms, and temporal relaxations slower than the molecular or supramolecular levels of organisation. The major functional consequence of this is that the qualitative behaviour of the system changes through the scaling of its spatio-temporal coordinates[9].

The statement made by von Bertalanffy[17] (quoted in Lloyd and Gilbert[46]) that "what are called structures are slow processes of long duration, functions are quick processes of short duration" is explained when one considers the functioning of cells or tissues at several simultaneous levels of organisation each one with characteristics space, $E_c$, and relaxation time, $T_r$. From distinct levels of organisation involved in microbial and plant cell growth, solute transport, energy transduction, neuron firing, and enzyme activity, $E_c$ and $T_r$ range from $10^{-11}$ to $10^{4}$ s and from $10^{-10}$ to $10^{-1}$ m[9,47].We have shown that $E_c$ and $T_r$ scale as a function of the level of organisation according to an allometric law, implying that both quantities grow exponentially. The exponent of the allometric relationship depicts the sensitivity of $E_c$ and $T_r$, characteristic of variations in the level of organisation. At lower levels of organisation, i.e., before the "transition point" (defined as the drastic change in slope of the allometric equation at spatial and temporal dimensions of micrometers and a few minutes), changes in the dynamics of processes given by their relaxation toward fluctuations are roughly threefold higher than the changes in spatial dimensions. Otherwise stated, temporal changes are more conspicuous than spatial changes. Further from this "transition point", where the system moves from microscopic to macroscopic order, the drastic increase in the slope of the allometric law suggests that structural patterns whose existence involve emergent macroscopic coherence imply that "essential" dynamic variables remain bounded or that attractors reduce to small ones. This behaviour "clamps" variables to a bounded (small) range of variation[9].

## MECHANISMS OF COHERENCE

### Bottom-Up Mechanisms

Most of the main functional properties of cells, such as energy transduction, solute transport, action potentials in neurons, macromolecules poly-merisation, and cell growth and division, are placed in $T_r$ ranges of 1 to 3 (seconds to several minutes on a logarithmic scale) and $E_c$ of -7 to -6 (around

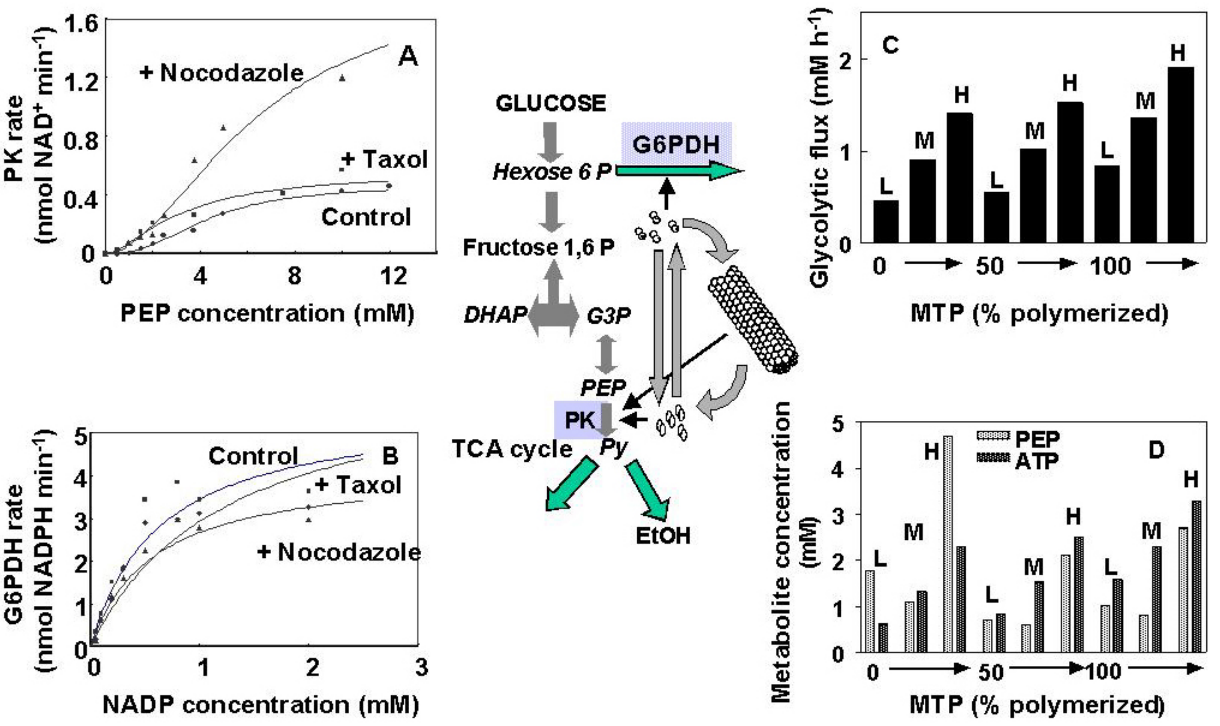

**FIGURE 4.** (Supra)molecularly organised environments may exert global modulation of metabolic networks. Cellular systems are able to show coherent as well as emergent properties that arise from dynamic organisation under homeodynamic conditions. Two important characteristics of microtubular networks make them likely candidates for the coherent synchronisation of intracellular dynamics[9,13,45,80,87]: 1) their nonlinear dynamics which, at some nonequilibrium state, might give rise to self-organisation and macroscopic structuring, and 2) their fractal or self-similar nature over a range of spatial length scales[9]. The figure shows the sensitivity of two coupled enzymatic reactions (pyruvate kinase/lactate dehydrogenase [PK/LDH], panel A and hexokinase/glucose-6-phosphate-dehydrogenase [HK/G6PDH], panel B) to the intracellular polymeric status of microtubules in permeabilized *Saccharomyces cerevisiae* cells in the presence of stabilizing (taxol) or depolymerizing (nocodazole) agents[81]. Plotted curves represent the best fit to the experimental data. Previous experimental analysis *in vitro* of coupled enzymatic reactions (PK/LDH, HK/G6PDH) in the presence of polymerised or nonpolymerised microtubular protein (MTP) showed that global activation of the flux may be achieved[81]. Another important piece of evidence showed that changes in the dynamics of tubulin assembly-disassembly may entrain the dynamics of enzymatic reactions[9,52]. The *in situ* kinetic parameters of PK or G6PDH (A, B) and their dependence on microtubules were taken into account in a mathematical model. This couples the dynamics of assembly-disassembly of microtubular protein (MTP) to the glycolytic pathway and branching to the tricarboxylic acid (TCA) cycle, ethanolic fermentation[82,83], and the pentose phosphate cycle (central scheme, green arrows). The mathematical model comprises 11 ordinary differential equations; 8 of them represent the intermediate concentration levels, whereas the other 3 describe the concentration of polymerised or nonpolymerised (GTP-bound) MTP and the oligomeric status of PK[9,51,81,82,83]. The polymeric status of MTP may globally modulate the glycolytic flux in a coherent manner (panels C, D). Indeed, the main results obtained show that the steady state glycolytic flux was globally increased under conditions in which 100% of the MTP was polymerised at either low (L = 1.93 m*M* min$^{-1}$), medium (M = 2.55 m*M* min$^{-1}$) or high (H = 3.15 m*M* min$^{-1}$) cellular glucose uptake rate (panel C). On the contrary, the flux through glycolysis was decreased when the bulk of MTP was depolymerised. Intermediary, although nonproportional, results were achieved when 50% of MTP was polymerised (panel C). In the presence of high or low levels of polymerised MTP the main rate-controlling steps of the glycolytic flux are the glucose uptake (positive), HK (positive), and the branch toward the pentose phosphate (PP) cycle (negative). Apparently, the negative control exerted by the PP pathway was less important at high than at low levels of polymerised MTP, in this way explaining the higher glycolytic fluxes attained[87]. Concomitantly with the global flux modulation, the steady-state values of metabolites also changed systemically (panel D). The fact that the alterations induced by the polymeric status of MTP on the metabolic network were properties of the integrated system was confirmed by varying individual kinetic parameters of enzymes (e.g., PK) coupled to MTP dynamics. In the latter case, only local changes in the level of metabolites (i.e., PEP) affected by the enzyme activity were observed[87,88].

micrometers)[9]. The temporal span corresponds to metabolic and epigenetic domains[8,46,48]. Molecular properties involved in those processes span $T_r$s of -6 to -10 (ms to ps) and $E_c$s of -8 to -10 (nanometers to angstroms). These are wide spatio-temporal spans. On these grounds, we have proposed that for molecular properties to extend their range of action to higher spatio-temporal dimensions, organising principles implying coherence in space and time must be invoked[9].

Relevant to the functional behaviour of cells or tissues are the mechanisms through which dynamic organisation is achieved under homeodynamic conditions. We have previously suggested that dynamically organised phenomena are visualised as being spatio-temporally coherent. Several mechanisms underlie this bottom-up coherence, that we briefly describe. Thus, for example, waves of second messengers or ions may arise through a combination of amplification in biochemical reaction networks and spreading through diffusion or percolation. Amplification may arise at instabilities in the dynamics of biochemical reactions by autocatalysis through allosteric or ultrasensitive mechanisms[13,49]. Under these conditions, transduction (sensitivity amplification) and coherence (spatio-temporal waves) mutually cooperate.

Dynamic supramolecular organisation of components of the cytoskeleton gives rise to sophisticated spatial organisation and intricate fractal geometry in cells. The cellular cytoskeleton fulfills all the requirements for self-organisation, i.e., they are open to fluxes of matter (proteins) and energy (GTP), and non-linearity is provided by autocatalysis during polymerisation. Similar considerations apply to the so-called dynamic instability that results in the catastrophic depolymerisation of microtubules[50]. We have offered an interpretation of microtubular dynamic instability in terms of an irreversible bistable transition. Thus, the dynamic coupling between changes in cytoskeleton organisation and of enzymatic reactions taking place concomitantly produces entrainment of one system by the other, in a global bistable switch (Fig. 4)[51,52,87].

Biochemically and thermodynamically, cellular metabolism may be represented as a set of catabolic and anabolic fluxes coupled to each other through energy-transducing events. In this framework, pathway stoichiometry constitutes a built-in autocatalytic source of nonlinear kinetics able to give rise to homeodynamic behaviour, i.e., both monotonic and periodic. Thus, the coordination of metabolic fluxes is an expression of the cell's dynamic organisation. Flux coordination is, in turn, involved in the regulation of cell growth. We have studied the processes of growth, division and sporulation in *S. cerevisiae*; subcellular structural remodelling can be related in this system to the degree of coupling between carbon and energy fluxes[9,53,54,55].

The ability of biological systems, either unicellular or multicellular, to exhibit rhythmic behaviour in the ultradian domain is a fundamental property because of its potential role as an inducer of bottom-up coherence, e.g., entrainment of cell division[8], or coordination of intracellular functions (top-bottom coherence: see below). In systems exhibiting chaos, many possible motions are simultaneously present. In fact, since the dynamics of a chaotic system traces a strange attractor in the phase space, in principle a great number of unstable limit cycles are embedded therein; each of these is characterised by a distinct number of oscillations per period[56,57]. Within the perspective of homeodynamics, biological systems exhibiting chaotic dynamics need only small perturbations of their parameters in order to select stable periodic outputs[58,59]. This characteristic facilitates dynamic motion of the system between attractors, i.e., *homeodynamics* (Fig. 3).

### Top-Bottom Mechanisms: Ultradian and Circadian Clocks

Recently, it has become evident that the circadian clock-control dominates the entire functioning of the organism in slowly growing lower eukaryotes as well as in some prokaryotes[48]. Circadian gating of the cell division cycle under daily alternation of light and dark, for example (Fig. 5), is well documented[46,48,84]. Circadian control of gene expression in the cyanobacteria *Synechococcus* has been reported[85].

It has been proposed that the ultradian clock has timing functions providing a time base for intracellular coordination[8]. In the latter sense, ultradian oscillations are, potentially, a coherence-inducer of the top-bottom type. The ultradian clock has multiple outputs, e.g., rhythms of respiration, adenine nucleotides, accumulating protein, enzyme concentration and activity, and it provides a time-frame for cell division (Fig. 6)[48,86]. Cycles of activity of energy-yielding processes are the consequences of timer-controlled alternating phases of high and low biosynthetic energy need[29,60,61]. Epigenetic ultradian oscillations with periods that range between 30 min and 4 h have been identified as playing a central timekeeping role in embryos and in lower eukaryotes under conditions of rapid growth (see ref. 46 for a review). Thus, in rapidly dividing organisms, the organisation of central metabolic processes (energy generation and biosynthetic pathways) requires a time-base given by phase-locking to the ultradian clock[48]. Limitation of the respiratory rate by ADP levels and their phase relationship take part of the ultradian clock mechanism (Fig. 6). Mitochondrial activities are determined by energy requirement on an epigenetic time scale rather than on a faster metabolic dynamic[8].

The ultradian clock has been interpreted as a forcing function in the differential equation for the slow variable in a mathematical model that basically represents a cell division cycle oscillator with a slow and a fast component[8,62]. The short-period (ultradian) clock exerts a dominant control of the cell division time, in both lower eukaryotes and in higher animal cells in culture[46].

Another model of the cell division cycle takes into account the coordination of macromolecular synthesis by cyclin-dependent kinases whose active forms are a complex of at least a kinase and a cyclin called maturation promoting factor (MPF)[48,63,64]. The latter model's homeodynamic behaviour shows three modes: as a steady state with high MPF activity, as a spontaneous oscillator, or as an excitable switch [63,64].

## CONCLUSIONS AND OUTLOOK

Dynamic systems exhibit different types of attractors either large or small. Large attractors are more likely to behave

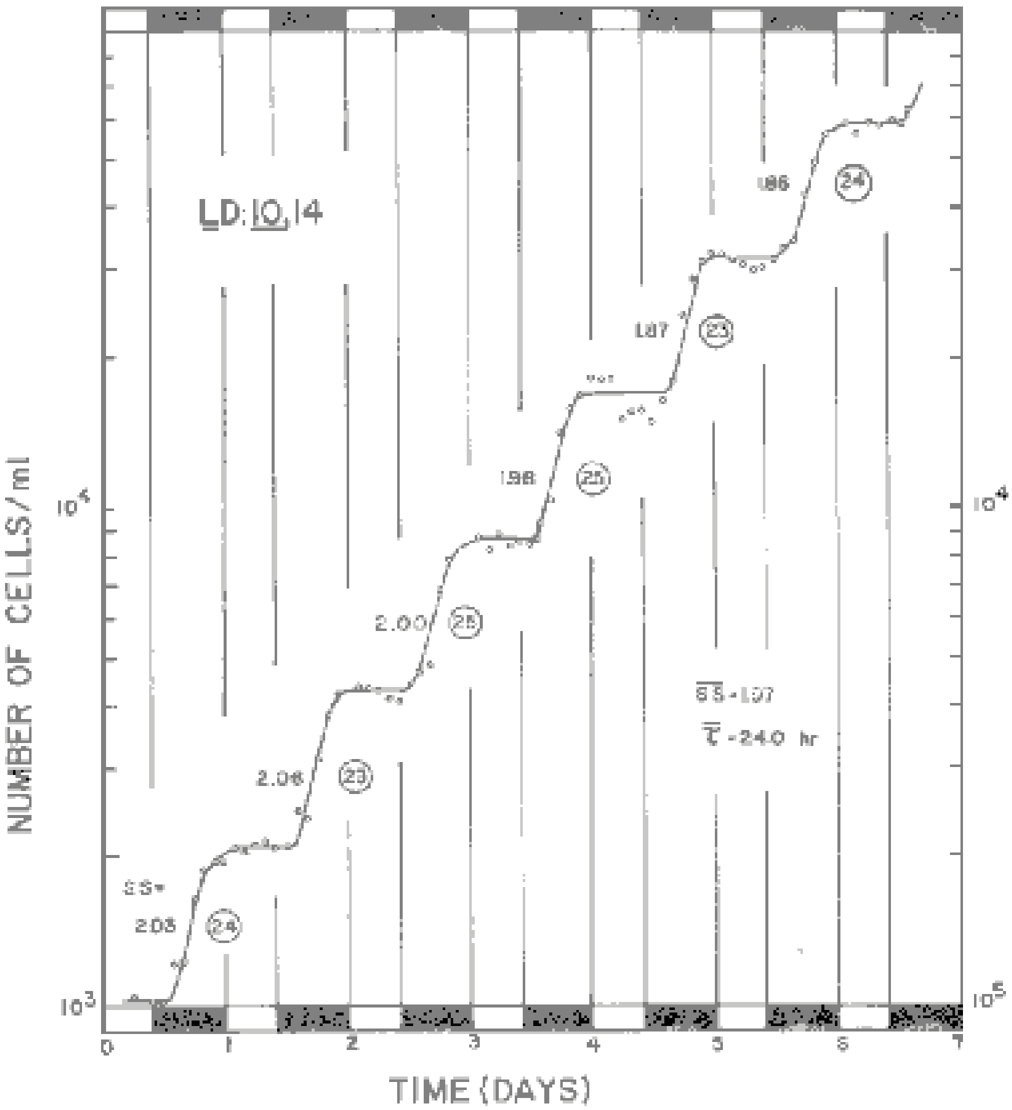

**FIGURE 5.** Circadian clock control of the cell division cycle in the algal flagellate *Euglena*. Entrainability is a key property of circadian rhythms according to which circadian rhythmicities can be synchronized by imposed diurnal light or temperature cycles to precise 24-h periods and can be predictably phase-shifted by single light and temperature signals.[26,35,48,84] The figure illustrates the entrainment of the cell division rhythm in populations of *Euglena gracilis* batch cultured photoautotrophically at 25°C by a full-photoperiod, diurnal light-dark (LD) cycle: 10, 14 light cycle. Step sizes (ss, ratio of number of cells per milliliter after a division burst to that just before the onsets of divisions) are given for successive steps; the estimated period (t) of each oscillation (intervals between successive onsets of divisions) is indicated by the encircled numbers (hours). The average period ('t) of the rhythm in the culture was almost identical to that (*T*) of the synchronizing LD cycle. Divisions were confined primarily to the main dark intervals, commencing at their onsets. A doubling cell number (ss @ 2.00) usually occurred every 24 h in this full-photoperiod LD cycle. (Reproduced from Edmunds, 1988, by permission of Springer-Verlag, New York.)

*homeodynamically* with respect to small attractors, because "essential" variables are likely to be subjected to a large range of variation rather than remaining clamped to small variations. Thus, we suggest, homeodynamics as a more general and all-embracing concept of which homeostasis becomes a special case, is better suited for describing the temporal structure of living systems. Under homeodynamic conditions, living systems spatio-temporally coordinate their functioning by essentially top-bottom or bottom-up mechanisms. The former are represented by circadian and ultradian rhythms with clock characteristics, whereas the latter emerge from the intrinsic, autonomous dynamics of the integrated mass-energy-information carrying networks that represent living systems.

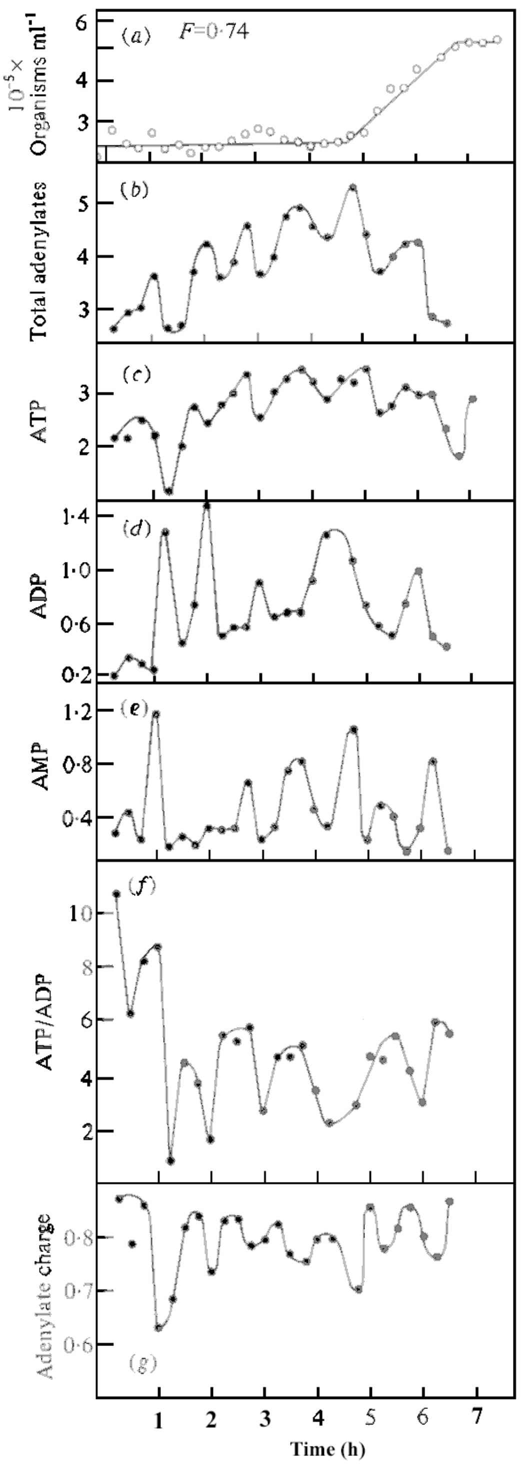

**FIGURE 6.** Cell-cycle-ultradian clock interactions in *Acanthamoeba castellanii*. In rapidly dividing organisms the organization of central metabolic processes (energy generation and biosynthetic pathways) requires a time-base, and the processes are phase-locked to a central oscillator, in this case the ultradian clock.[48] The figure shows changes in adenine nucleotide pool levels and adenylate charge values in a synchronously dividing culture of *A. castellanii* ($\tau$ = 69 min at 30°C).[86] The synchronous culture contained 10% of the exponentially growing population. Adenine nucleotides were measured in 1 ml samples withdrawn at 15 min intervals. (a) Cell numbers and synchrony index, *F*. Adenylate concentrations are expressed as nmol mL$^{-1}$ culture. (Reproduced from Edwards and Lloyd, 1978, by permission of The Society for General Microbiology.)

Globally organised complexity is brought forth by the cross-talk between these two opposing, but complementary, flows of information. In this realm, gaps in our knowledge are still large, but pointing to where the trend of our efforts should be directed.
